# Fall characteristics among elderly populations in urban and rural areas in Korea

**DOI:** 10.1097/MD.0000000000023106

**Published:** 2020-11-13

**Authors:** Myeongkyu Kim, Misoo Chang, Eunwoo Nam, Seul Gi Kim, Sung-il Cho, Dong Hee Ryu, Sin Kam, Bo Youl Choi, Mi Jung Kim

**Affiliations:** a Department of Rehabilitation Medicine, Hanyang University, College of Medicine; b Research Coordinating Center, Konkuk University Medical Center; c Section of Pharmacoepidemiology, Hanyang University Hospital for Rheumatic Disease; d Graduate School of Public Health and Institute of Health and Environment, Seoul National University, Seoul; e Department of Preventive Medicine, Daegu Catholic University School of Medicine; f Department of Preventive Medicine, School of Medicine, Kyungpook National University, Daegu; g Department of Preventive Medicine, Hanyang University, College of Medicine, Seoul, Republic of Korea.

**Keywords:** elderly, fall, rural, urban

## Abstract

Falling is one of the leading causes of injury among elderly populations. As the population over 65 years old increases, medical costs due to falling will also increase. Urban and rural areas have different fall characteristics, and research into these differences is lacking.

A survey was conducted on 2012 people over 60 years old between September 1, 2015, to October 12, 2015. Guro-gu (Seoul), Yeongdeungpo-gu (Seoul), and Jung-gu (Daegu) were classified as urban areas and included 1205 of the study participants. Dalseong-gun (Daegu) and Yangpyeong-gun (Gyeonggi-do) were categorized as rural areas and included 807 participants. The survey included questions about fall history, cause, season and time of recent falls, and external conditions associated with recent falls, like floor or ground materials and shoe types.

Rural respondents were older than urban respondents (*P* < .001) but did not differ significantly in gender proportion (*P* = .082). Fall history over the past year was not different between the 2 regions (*P* = .693), but lifetime fall history was greater among rural respondents (*P* < .001). Only 5.1% of all respondents had undergone fall-prevention education. A slippery floor was the most common cause of falls in both regions, but there was a significant difference in pattern of fall causes (*P* < .001). Falls were more frequent in the summer, spring, and the afternoon in urban areas, and in the summer, autumn, and the morning in rural areas. Cement and asphalt were the most common ground materials at the time of falls in both regions, but rural respondents had higher fall rates when walking on soil and when wearing slippers.

A fall-prevention program that reflects the characteristics and differences of falls in urban and rural areas should be developed and used to effectively prevent falling among elderly people.

## 1 Introduction

A fall is defined by the World Health Organization (WHO) as an incident that leads to a person unintentionally coming to rest on the ground, floor, or other lower level.^[1]^ Falls can happen at any age, but falls are more common among the elderly than young people. In addition, elderly people are at a greater risk of injury when they fall.^[2]^ A study of the 2014 Behavioral Risk Factor Surveillance System (BRFSS) survey found that 28.7% people 65 years or older in the United States (29.0 million people) experienced a fall during the past 12 months, and 37.5% of them (7.0 million people) had injuries that require medical treatment.^[3]^

Falls can cause nonlife-threatening injuries, such as dislocations, sprains, cuts, and abrasions, but hemorrhage from head injuries and fractures can be fatal.^[4,5]^ Falls are responsible for 98% of hip fractures,^[6]^ and the 1-year mortality rate after hip fracture is 8.4%-36%, higher than nonhip fracture groups or community-living control populations.^[7]^ A survey of 409 community-dwelling elderly people aged 65 years or older in Montreal, conducted from May 1987 to October 1988, reported 197 falls, 91 fall-related injuries (46.2%), and 3 hip fractures out of a total of 5 fracture cases.^[8]^ Data based only on elderly people who live in assisted-living facilities indicate lower rates of fall-related injuries, but not fewer hip fractures. On the basis of fall data from 469 elderly people living in residential-care facilities in Sweden in 1997 over a single year, fall-related injuries occurred in 1 quarter of the 865 falls and 18 hip fractures among 24 total fractures were reported.^[4]^

The number of elderly people and their proportion of the total population is increasing worldwide. The elderly population (≥65 years) in Europe is expected to increase from 129.6 million (17.4%) in 2015 to 196.8 million (27.8%), and from 54.6 million (15.1%) in 2015 to 94.6 million (21.4%) in 2050 in North America. The same trend is seen in other continents, including Asia and Africa.^[9]^ As the elderly population grows, medical expenses due to falls are becoming a great social burden. In the United States, a survey of seniors aged 65 years and older reported that the direct medical cost from falls was 616.5 million dollars in 2012 (average cost per fatal fall: 25,487 dollars) and 637.2 million dollars in 2015 (average cost per fatal fall: 26,340 dollars). The direct medical cost from non-fatal falls was 30.3 billion dollars in 2012 (average cost per nonfatal fall: 9463 dollars) and 31.3 billion dollars in 2015 (average cost per nonfatal fall: 9780 dollars).^[10]^ Total and per-fall costs are increasing; thus, efforts are needed to understand and reduce falls in older people.

To date, most research on falling has focused on urban residents, and studies on rural areas have only been published recently. Falling incidence and fall-related cost in rural elderly population has been studied but separately from urban populations.^[11,12]^ A study by Park et al^[13]^ about rural region revealed that female gender and older age are risk factors for fall. In a study using data from 16,393 people over 65 in China, Zhang et al^[14]^ compared the fall characteristics of rural and urban areas. Fall incidence was higher in rural area, and hospitalization rate due to fall was higher in urban area in this study. In addition, more fall occurred in urban roads and rural yards. Yoo et al^[15]^ showed dizziness, walking discomfort, and fear of falling were influencing factors for fall in rural area, and stroke, visual impairment, and the fear of falling in urban area using data from 534 elderly people in Korea.

As there are insufficient data comparing the fall characteristics of rural and urban areas, research comparing 2 regions as an effort to understand and reduce falls in each region is still meaningful. This study compares fall characteristics between urban and rural areas using data from a large-scale survey carried out in Korea, and provides basic information that is not covered in other studies, such as frequency, cause, time, and shoes worn.

## 2 Methods

### 2.1 Participants

This study is based on survey results from people over 60 years old who visited a public health center in their district. The survey was completed by 2012 people living in 5 regions of Korea from September 1, 2015, to October 12, 2015. Among the 5 regions, Guro-gu (Seoul, n = 401), Yeongdeungpo-gu (Seoul, n = 404), and Jung-gu (Daegu, n = 400) were classified as urban areas, and Dalseong-gun (Daegu, n = 400) and Yangpyeong-gun (Gyeonggi-do, n = 407) were classified as rural areas. Table 1 summarizes the differences between population density and land use in urban and rural areas.^[16]^

**Table 1:** Characteristics of survey areas (2014).

	Urban area			Rural area	
	Guro-gu (Seoul)	Yeongdeungpo-gu (Seoul)	Jung-gu (Daegu)	Yangpyeong-gun (Gyeonggi-do)	Dalseong-gun (Daegu)
Population density (people per square mile)	58,844.5	44,498.6	29,204.7	315.1	1123.5
Percentage of farmland, orchard, ranch and forest in the total area (%)	21.4	0.6	0.0	88.0	79.3
Number of senior welfare facilities per 1000 elderly people	2.73	2.84	2.51	11.97	9.46

### 2.2 Questionnaire

The questionnaire, written in Korean, included several questions to characterize falls. It excluded questions about personally identifiable information, and only gender and age were recorded. Fall-experience questions included lifetime fall history, past 1-year fall history, experience with fall-prevention education, cause of fall, season and time of day, floor or ground materials, and shoe type.

If there was more than one event that fitted the definition of fall as described above after becoming an adult, it was recorded in the lifetime fall history category. Slight fall that did not require medical treatment was also included. The past 1-year fall history item is to be answered only if fall has occurred in the past year. Those who have participated in fall-prevention education by government, public institutions, hospitals, or religious facilities were required to respond in fall-prevention education category.

Participants in the study chose the cause of fall among slippery floor, ankle spraining caused by sudden inversion of ankle due to uneven floor when stepping on the foot, stumbling over door still, collision with people or objects unexpectedly, dizziness, steep slope, stumbling over stairs, dark lighting, and impaired eyesight. If participants could not select appropriate item, they were asked to respond to the “other” item. The season that fall occurred in the past year was selected among spring (March to May), summer (June to August), autumn (September to November), and winter (December to February). Floor or ground material at the time of the fall was chosen among cement or asphalt, soil, linoleum, tile, ice, and cannot recall. Shoe type was selected from running shoes, bare foot, slippers, dress shoes, and cannot recall.

Only willing volunteers completed surveys, and informed consent was obtained after providing potential participants with sufficient study information. All participants were additionally informed that they could stop their participation at any time. This study and the questionnaire were approved by the Hanyang University Medical Center Institutional Review Board (No. HYI-15–008-2).

### 2.3 Statistical analyses

This study analyzed differences in fall characteristics between urban and rural areas using descriptive statistical methods. Handwritten survey responses were transcribed into a database by 6 researchers who followed consistent data-entry rules. The Chi-square test was used to compare demographic data such as age and gender between the urban and rural areas. To compare the characteristics of urban and rural falls, differences were estimated using the Chi-square test. Statistical analyses were carried out using SPSS software ver. 21.0 (IBM, Armonk, NY).

## 3 Results

### 3.1 Demographic characteristics

Of the 2012 participants in this study, 1205 lived in urban area and 807 lived in rural areas. Among urban and rural residents, respectively, 36.0% and 23.4% were in their 60s, 46.1% and 52.4% were in their 70s, and 17.9% and 24.2% were 80 or over. Rural residents were significantly older than urban participants (*P* < .001), and 63.4% and 67.0% of urban and rural area residents, respectively, were women (*P* = .082; nonsignificant). Lifetime fall history was significantly higher in rural areas (78.9%) than in urban areas (69.3%; *P* < .001). However, fall history during the 1 year before the survey was similar between areas (*P* = .693). Ninety-eight people in the urban areas (8.1%) and only 4 people in rural areas (0.5%) underwent fall-prevention education at any time in the past, which is a significant difference (*P* < .001; Table 2). For more detailed demographic data of participants in the study, refer to the results of Lee et al,^[17]^ who presented the fall rate of the elderly in Korea based on the survey conducted in the present study.

**Table 2:** Demographic characteristics and prior fall-prevention education in urban and rural areas.

	Urban area^∗^ (%) (n = 1205)	Rural area^†^ (%) (n = 807)	Total (%) (N = 2012)
Age, y^‡^			
60–69	434 (36.0)	189 (23.4)	623 (31.0)
70–79	555 (46.1)	423 (52.4)	978 (48.6)
≥80	216 (17.9)	195 (24.2)	411 (20.4)
Gender			
Male	439 (36.4)	263 (32.6)	702 (34.9)
Female	764 (63.4)	541 (67.0)	1305 (64.9)
No answer	2 (0.2)	3 (0.4)	5 (0.3)
Lifetime fall history^‡^			
Yes	835 (69.3)	637 (78.9)	1472 (73.2)
No	370 (30.7)	170 (21.1)	540 (26.8)
Fall history (past one year)			
Yes	393 (32.6)	270 (33.5)	663 (33.0)
No	812 (67.4)	537 (66.5)	1349 (67.1)
Fall-prevention education^‡^			
Yes	98 (8.1)	4 (0.5)	102 (5.1)
No	1078 (89.5)	769 (95.3)	1847 (91.8)
No answer	29 (2.4)	34 (4.2)	63 (3.1)

### 3.2 Causes of falls

Among the 663 respondents who experienced a fall in the past year, the most common cause in both urban (32.6%) and rural (42.1%) areas was a slippery surface, according to multiple-choice response. In urban areas, stumbling on a door sill (14.0%) and ankle sprain (13.6%) were the next common causes. In rural areas, ankle sprain (11.6%), dizziness (10.6%), and a steep slope (7.7%) were the next most common causes. Differences in cause of fall were significant between urban and rural areas in a Chi-square test (*P* < .001; Table 3).

**Table 3:** Fall causes in urban and rural areas over the past year (duplicate responses included).

	Urban area^∗^ (%)	Rural area^†^ (%)	Total (%)
Fall causes^‡^			
Slippery floor	154 (32.6)	131 (42.1)	285 (36.4)
Spraining ankle	64 (13.6)	36 (11.6)	100 (12.8)
Stumbling over door sill	66 (14.0)	23 (7.4)	89 (11.4)
Collision	49 (10.4)	23 (7.4)	72 (9.2)
Dizziness	28 (5.9)	33 (10.6)	61 (7.8)
Steep slope	21 (4.5)	24 (7.7)	45 (5.8)
Stumbling over stairs	28 (5.9)	9 (2.9)	37 (4.7)
Dark lighting	17 (3.6)	9 (2.9)	26 (3.3)
Impaired eyesight	6 (1.3)	4 (1.3)	10 (1.3)
Other	39 (8.3)	19 (6.1)	58 (7.4)

### 3.3 Season and time of fall

The 663 respondents who experienced a fall in the past year were asked about the month and season their fall occurred. Falls occurred most frequently in the summer (June--August, 29.8%) and spring (March--May, 29.0%) in urban areas, and in the summer (27.8%) and autumn (26.3%) in rural areas. Seasonal fall rates were statistically different between the 2 regions (*P* = .009). Regarding time of day, 55.2% of falls occurred in the afternoon (12:00 pm--18:00 pm) in urban areas, and 45.9% of falls occurred in the morning (06:00 am--12:00 pm) in rural areas. The fall rate according to time of day was significantly different between the 2 areas (*P* < .001; Table 4).

**Table 4:** Season and time of falls in urban and rural areas over the past year.

	Urban area^∗^ (%) (n = 393)	Rural area^†^ (%) (n = 270)	Total (%) (n = 663)
Season^‡^			
Spring (March–May)	114 (29.0)	58 (21.5)	172 (25.9)
Summer (June–August)	117 (29.8)	75 (27.8)	192 (29.0)
Autumn (September-November)	64 (16.3)	71 (26.3)	135 (20.4)
Winter (December–February)	98 (24.9)	66 (24.4)	164 (24.7)
Time^‡^			
Dawn (00:00–06:00)	24 (6.1)	9 (3.3)	33 (5.0)
Morning (06:00–12:00)	98 (24.9)	124 (45.9)	222 (33.5)
Afternoon (12:00–18:00)	217 (55.2)	118 (43.7)	335 (50.5)
Night (18:00–24:00)	42 (10.7)	12 (4.5)	54 (8.1)
No answer	12 (3.1)	7 (2.6)	19 (2.9)

### 3.4 Floor or ground material and shoes type

As above, the 663 people who experienced a fall in the past year were asked about the floor or ground material where they fell. Roads made of cement or asphalt were the most common ground materials at the time of the fall in both urban (38.7%) and rural (37.8%) areas. Soil (20.1%), tile (15.3%), linoleum (13.7%), and ice (5.1%) were the next common materials in urban areas, while soil (30.0%), linoleum (13.0%), tile (10.0%), and ice (4.1%) were the next common in rural areas; the differences between urban and rural areas were significant (*P* = .008). When asked about shoes worn at the time of the fall, 58.0% of urban and 45.2% of rural respondents reported running shoes. Other responses were barefoot (18.1%), slippers (11.2%), and dress shoes (4.6%) in urban areas, and slippers (22.6%), barefoot (16.3%), and dress shoes (3.3%) in rural areas, and the difference between urban and rural areas was significant (*P* < .001; Table 5).

**Table 5:** Floor or ground materials and shoe type at the time of falling in urban and rural areas over the past year.

	Urban area^∗^ (%) (n = 393)	Rural area^†^ (%) (n = 270)	Total (%) (n = 663)
Floor or ground materials^‡^			
Cement, asphalt	152 (38.7)	102 (37.8)	254 (38.3)
Soil	79 (20.1)	81 (30.0)	160 (24.1)
Linoleum	54 (13.7)	35 (13.0)	89 (13.4)
Tile	60 (15.3)	27 (10.0)	87 (13.1)
Ice	20 (5.1)	11 (4.1)	31 (4.7)
Cannot recall	13 (3.3)	2 (0.7)	15 (2.3)
Other	11 (2.8)	12 (4.4)	23 (3.5)
No answer	4 (1.0)	0 (0.0)	4 (0.6)
Shoe type^‡^			
Running shoes	228 (58.0)	122 (45.2)	350 (52.8)
Barefoot	71 (18.1)	44 (16.3)	115 (17.4)
Slippers	44 (11.2)	61 (22.6)	105 (15.8)
Dress shoes	18 (4.6)	9 (3.3)	27 (4.1)
Cannot recall	2 (0.5)	5 (1.9)	7 (1.1)
Other	26 (6.6)	28 (10.4)	54 (8.1)
No answer	4 (1.0)	1 (0.4)	5 (0.8)

## 4 Discussion

In this study, we compared fall characteristics between urban and rural areas over the same time period, and found significant differences in various categories. Rural participants were statistically older than urban participants, and proportion of female respondents was insignificantly higher in rural subset. Differences in age and gender composition are a result of differences in demographic structure between urban and rural areas. Population data for 2014 showed that people over 60 years comprised 15.7% of urban communities (Guro-gu, Yeongdeungpo-gu, and Jung-gu) and 19.8% of rural communities (Dalseong-gun and Yangpyeong-gun).^[16]^

We did not find a statistical difference in the fall rate over the previous year between urban and rural areas, which is in line with other studies. A 2014 study of Australians over 50 years found no difference in fall hospitalizations, fall mortality, or fall-related injuries between city and rural participants.^[11]^ However, we found that lifetime fall history was higher in rural areas, perhaps because the average age of our rural participants was higher than of urban participants. Also, it is thought that there are many people in rural areas who fall, including those younger than 60 years, due to environmental differences, and this is one of the causes for higher lifetime fall history in rural areas.

People in urban and rural areas typically use different medical services after falling because of disparities in medical accessibility. Byles et al^[18]^ found that fall patients were more likely to be treated by general practitioners and specialists in urban areas, while nonurban patients usually accessed community services and alternative health practitioners. Therefore, it is particularly important to provide good fall-prevention education in rural areas. However, as summarized in Table 1, despite rural areas having higher ratios of elderly welfare facilities to senior residents, fewer rural respondents had received fall-prevention education than urban respondents.

The effectiveness of fall-prevention interventions has been demonstrated in a number of studies. A 27-month survey of people over 65 years old in Denmark, including 12,905 people who underwent fall-prevention education and 11,460 control participants showed that all types of fracture injuries were reduced in the intervention group, especially lower-extremity fractures, which decreased by 33%.^[5]^ Hill-Westmoreland et al^[19]^ showed that the fall rate was 4% lower among intervention groups compared with control groups in a meta-analysis of 12 studies. In addition, the effect was stronger when risk modification and comprehensive risk assessment were performed simultaneously, rather than exercise alone.^[19]^ Therefore, fall-prevention efforts are a necessary part of an educational program, which should also address issues elucidated by our above-mentioned results, including causes, characteristics, and risk factors for urban and rural populations.

We found that slippery floors were the leading cause of falls in both urban and rural areas, which is consistent with findings from the United States and China.^[20,21]^ However, we also found that slipping rates were higher in rural areas than cities and stumbling on door sills was more common in urban areas. These findings should be taken into account when designing public buildings or facilities related to elderly citizens. Also, because falls caused by collisions are more common in urban areas, this should be emphasized in urban fall-prevention education.

Both urban and rural participants in this study experienced the most falls during summer, a finding that differed from other studies. Bulajic-Kopjar^[22]^ analyzed 10,992 fall-related fractures in Norwegian 65 years and older, and found that the risk of falling was greater during the colder months for people 65 to 79 years old and for people over 80 years. Furthermore, the fractures that occurred during the winter were mostly caused by ice and snow, and the rate of hip and arm fractures also increased.^[22]^ Caberlon and Bós^[23]^ studied people over 60 years old in Rio Grande do Sul of Brazil, a temperate region, and also found that the largest number of falls occurred during winter (26.8%), and 34% of all fractures occurred in winter as well. In this study, falls are concentrated in summer, when outdoor activity increased. However, almost 25% of falls still occur in winter, and winter falls usually carry the higher injury risk; thus, interventions to prevent winter falls are still very important.

This is the first study to compare the time of fall occurrence between urban and rural areas. As summarized in Table 4, falls were concentrated in the afternoon in urban areas, and in the morning in rural areas. The low fall rates at night in urban and rural areas seem to be related to the low proportion of low lighting in the causes of falls. However, the smaller number of night-time falls is likely partially attributed to less outdoor activity and does not necessarily indicate adequate lighting. Although some researchers expected better lighting to minimize falls,^[24]^ most studies have been performed only in indoor environments,^[25]^ and further research is needed on the relationship between lighting and falls.

We also evaluated whether there was a difference in the floor or ground materials during falls in urban and rural areas. Both had the highest number of falls on cement or asphalt. However, in rural areas, the rate of falls on soil was higher than in urban areas, possibly indicating that falls in rural areas frequently happen while performing farming activities, which is consistent with previous studies.^[21]^ In the United States, a study of people 65 years and older found the highest rate of falls occurred while working around the houses or yard (29%).^[20]^ This suggests that, for rural elderly people, particular attention should be paid to falls that occur during economic activities, such as farming, and to falls that occur on ordinary roads. Previously, there were few data about floor or ground materials with regard to elderly fall risk. One study of students on a playground found that the fall rate was highest on asphalt.^[26]^

Most urban and rural respondents in our study reported wearing running shoes at the time of their fall, but the proportion who fell in slippers was higher among rural people than urban people. Sherrington and Menz^[27]^ found that, among 95 patients with hip-fracture patients, the largest number was wearing slippers when they fell. Therefore, special attention in rural areas should be paid to safety while wearing slippers. They also found that 75% of survey participants suffered falls when wearing shoes with suboptimal features such as no fixation or an excessively flexible heel or sole.^[27]^ Recently, shoes that sound an alarm when approaching an obstacle have been developed to reduce falls.^[28]^ The development of these technologies will also play an important role for the prevention of falls in the future, but now fall-prevention education, including how to wear shoes properly, should also be emphasized.

When analyzing only the case of falling with slippers, the results showed that there seemed to be a region-dependent relationship between the floor or ground materials and the shoes. There were 44 cases of falling with slippers in urban areas (cement 22.7%, tile 61.4%, soil 11.4%, and others 4.5%), and 61 cases in rural areas (cement 37.7%, tile 24.6%, soil 23.0%, and others 14.8%), and there was a significant difference between 2 regions (*P* = .001). In particular, the proportion of people falling on the tile wearing slippers in urban areas was relatively higher than rural areas. It seems that this is because elderly people usually wear slippers in tiled places in urban settings, while they live in more diverse environments wearing slippers in rural areas.

This paper has several limitations. First, we only included elderly subjects who were able to visit public health centers; thus, we did not collect data from people who were severely injured by falling. This sampling error may cause study participants to lose their representativeness of the elderly group in the region. Second, because this is a retrospective and cross-sectional study with a survey design, a recall bias may have been present for many items about past falls. In particular, a person with recurrent fall experiences may have inaccurate memories about each fall. Future research should include further analyses of fall characteristics between urban and rural areas with larger patient sample for more robust results.

## 5 Conclusion

In this study, we compared fall characteristics between urban and rural areas. The rural participant group was older than the urban group. Although the previous-year fall rate was not different between the 2 groups, lifetime fall history was higher in the rural group. Falls most frequently occurred on slippery floors in both areas. Falls occurred most often in summer and spring in cities, and in summer and autumn in rural areas. Fall occurred most often in the afternoon in urban areas and in the morning in rural areas. Falls on soil and falls in slippers were more common in rural areas than urban areas. Because the proportion of elderly people who received fall-prevention education was low in both urban and rural areas, relevant policies to institute intervention programs that reflect the fall characteristics of urban and rural areas are immediately necessary.

## Author contributions

MK and MJK were involved in drafting and revising the manuscript. MC and EN contributed significantly to statistical analyses. Data collection and management were handled by SGK and SC in Seoul, DHR and SK in Daegu, and BYC in Yangpyeong-gun. All authors made substantial contributions to the conception, design, and data acquisition and analyses for this study. All authors read and approved the final manuscript.

**Conceptualization:** Myeongkyu Kim, Mi Jung Kim.

**Data curation:** Myeongkyu Kim, Mi Jung Kim.

**Formal analysis:** Myeongkyu Kim, Misoo Chang, Eunwoo Nam, Mi Jung Kim.

**Funding acquisition:** Mi Jung Kim.

**Investigation:** Seul Gi Kim, Sung-il Cho, Dong Hee Ryu, Sin Kam, Bo Youl Choi.

**Methodology:** Myeongkyu Kim, Mi Jung Kim.

**Project administration:** Misoo Chang, Eunwoo Nam, Seul Gi Kim, Sung-il Cho, Dong Hee Ryu, Sin Kam, Bo Youl Choi.

**Resources:** Seul Gi Kim, Sung-il Cho, Dong Hee Ryu, Sin Kam, Bo Youl Choi.

**Software:** Myeongkyu Kim, Misoo Chang, Eunwoo Nam, Seul Gi Kim, Sung-il Cho, Dong Hee Ryu, Sin Kam, Bo Youl Choi.

**Supervision:** Misoo Chang, Eunwoo Nam.

**Writing – original draft:** Myeongkyu Kim.

**Writing – review & editing:** Myeongkyu Kim, Mi Jung Kim.
